# Concomitant Exposure to Ovalbumin and Endotoxin Augments Airway Inflammation but Not Airway Hyperresponsiveness in a Murine Model of Asthma

**DOI:** 10.1371/journal.pone.0098648

**Published:** 2014-06-26

**Authors:** John Mac Sharry, Karim H. Shalaby, Cinzia Marchica, Soroor Farahnak, Tien Chieh-Li, Susan Lapthorne, Salman T. Qureshi, Fergus Shanahan, James G. Martin

**Affiliations:** 1 Meakins Christie Laboratories, Department of Medicine, McGill University, Montreal, Canada; 2 Alimentary Pharmabiotic Centre, School of Medicine, University College Cork, National University of Ireland, Cork, Ireland; Centre de Recherche Public de la Santé (CRP-Santé), Luxembourg

## Abstract

Varying concentrations of lipopolysaccharide (LPS) in ovalbumin (OVA) may influence the airway response to allergic sensitization and challenge. We assessed the contribution of LPS to allergic airway inflammatory responses following challenge with LPS-rich and LPS-free commercial OVA. BALB/c mice were sensitized with LPS-rich OVA and alum and then underwent challenge with the same OVA (10 µg intranasally) or an LPS-free OVA. Following challenge, bronchoalveolar lavage (BAL), airway responsiveness to methacholine and the lung regulatory T cell population (Treg) were assessed. Both OVA preparations induced BAL eosinophilia but LPS-rich OVA also evoked BAL neutrophilia. LPS-free OVA increased interleukin (IL)-2, IL-4 and IL-5 whereas LPS-rich OVA additionally increased IL-1β, IL-12, IFN-γ, TNF-α and KC. Both OVA-challenged groups developed airway hyperresponsiveness. *TLR4*-deficient mice challenged with either OVA preparation showed eosinophilia but not neutrophilia and had increased IL-5. Only LPS-rich OVA challenged mice had increased lung Tregs and LPS-rich OVA also induced *in vitro* Treg differentiation. LPS-rich OVA also induced a Th1 cytokine response in human peripheral blood mononuclear cells.We conclude that LPS-rich OVA evokes mixed Th1, Th2 and innate immune responses through the TLR-4 pathway, whereas LPS-free OVA evokes only a Th2 response. Contaminating LPS is not required for induction of airway hyperresponsiveness but amplifies the Th2 inflammatory response and is a critical mediator of the neutrophil, Th1 and T regulatory cell responses to OVA.

## Introduction

Allergen exposure is well recognized to participate in the induction of asthma. [1] Since the pioneering studies of Mosmann and colleagues, distinguishing two T helper subsets as Th1 and Th2 types [2], extensive studies on model systems of asthma have implicated a Th2 biased immune response in the pathogenesis of the disease. Over-expression of Th2 cytokines has also been confirmed in human asthmatic tissues and in induced sputum. [3], [4] Epidemiological studies have revealed a protective effect of large family size on the development of asthma, triggering the idea of the hygiene hypothesis in the protection against asthma. [5] Living in a rural environment with exposure to farm animals early in life was also shown to be protective against allergy and asthma. [6] High levels of endotoxin associated with this environment have been suggested to modulate innate immunity via Toll-like receptors (TLRs) and to promote the maturation of an immune system that is less predisposed to Th2 responses to innocuous aeroallergens. [7].

The complex mechanisms leading to Th2 immune responses to sensitization with allergen have been dissected using murine models that readily develop several of the characteristics of asthma, including eosinophilic inflammation and airway hyperresponsiveness following sensitization and challenge with allergen. [8]–[11] Dendritic cell processing of antigen and CD4+ T cell activation is the principal mechanism by which airway inflammatory responses to allergen are initiated. [12] Dendritic cell derived cytokines are key in determining the nature of the T cell response, whether Th1 or Th2 [12], Treg or Th17. [13] Other factors, such as cysteinyl-leukotriene production by dendritic cells [14] as well as basophil, mast cell and T cell derived cytokines may also alter the airway milieu in a way that favours Th2 differentiation. [15]–[17].

Innate immune mechanisms are required for dendritic cell priming to occur [12] and to cause effective Th2 responses to sensitization with ovalbumin. [18] Commercial OVA is known to be LPS-rich and the contamination appears to be of sufficient magnitude to affect allergic responses. [19] Previous studies have examined the effect of the contaminating LPS in ovalbumin or addition of exogenous LPS on the allergic sensitization process (whether intraperitoneal or intranasal) as well as subsequent allergen challenge. [18]–[22] Collectively, these studies have shown that the contaminating LPS and TLR4 signaling are necessary for priming pro-inflammatory T helper cell responses to inhaled ovalbumin and that the level of LPS exposure in conjunction with ovalbumin determines whether a Th2, Th1, Th17 and/or T regulatory cell response is elicited. [18], [20]–[23] In contrast, Watanabe *et al*. reported that the contaminating LPS in commercial ovalbumin preparations inhibited the development of allergic airway disease, though it is not clear whether this effect may have been related to the systemic co-exposure to LPS during sensitization rather than the pulmonary exposure during challenge. [19], [24] What, if any, effect the contaminating LPS in commercial ovalbumin preparations has specifically in relation to the secondary allergen exposure or “challenge” in animals which have been equivalently sensitized has not been examined to date. Furthermore, unlike intranasal ovalbumin sensitization, intraperitoneal sensitization with the adjuvant aluminum hydroxide in conjunction with ovalbumin has been shown to activate dendritic cells via the nucleotide-binding domain, leucine-rich repeat containing protein family, pyrin domain containing 3 (NLRP3) [25], bypassing the need for TLR-4 activation by LPS [21] in the induction of allergic airway disease. However, it is not known whether the contaminating LPS may still modulate the airway inflammatory response, even if it is not required for the manifestation of experimental asthma in these so-called “alum-based” allergy models. The aim of the present study was thus to contrast the airway responses to OVA by comparing LPS-rich and LPS-free OVA specifically during allergen challenge in a murine model of allergic asthma. For this purpose we assessed lung mechanical responses to methacholine challenge and the inflammatory response evoked by OVA. We also assessed the degree to which regulatory T cells were induced in response to OVA challenge and the effects of the TLR4 inhibitor, TAK242, on *in vitro* Treg induction in splenocytes. *TLR4*-deficient mice were also used to evaluate the role of TLR4 *in vivo* in the modulation of airway inflammation by contaminating LPS. Finally we measured the responses of peripheral blood mononuclear cell responses of normal human volunteers to incubation with LPS-free and LPS-containing OVA to assess the role of immune effector cells on cytokine responses to LPS-rich OVA.

## Methods

### Assessment of endotoxin levels in OVA

Endotoxin levels were assessed in two sources of OVA; Sigma Grade V OVA (LPS-rich) Sigma, St Louis MO or Dorset, UK and Endograde-OVA (LPS-free) (Hyglos Gmbh, Regensburg, Germany) using two methods. The first was the Limulus Amebocyte Lysate Pyrogent Single Test Vials (Lonza, Walkersville, MD, USA) with a maximum sensitivity of 0.06 EU/ml, this was used to qualitatively test for the presence of LPS in both samples. Five different concentrations of Sigma and Hyglos OVA were tested for the presence of LPS (10 µg/ml, 1 µg/ml, 100 ng/ml, 10 ng/ml, 1 ng/ml). LAL reagent water was used as a negative control and 0.25 ml of each sample was added to the test vials containing lysates from washed amebocytes, following the manufacturer’s instructions. A positive outcome was detected as a firm gel formation in the test vial after inversion.

To obtain a more quantitative assessment of endotoxin levels we used the ToxinSensor Chromogenic LAL Endotoxin Assay Kit (GenScript, Piscataway, NJ, USA) with a sensitivity range of 0.005–1 EU/ml. Concentrations of Sigma-OVA ranging from 0.1 ng/ml to 100 ng/ml, and five concentrations of Hyglos-OVA ranging from 10 µg/ml to 100 ng/ml were used to evaluate LPS content. First, 100 µl of LAL was added to 100 µl of each sample followed by an incubation period of 45 minutes at 37°C. Following the incubation period, 100 µl of chromogenic substrate was added to each sample vial followed by 6 minutes incubation at 37°C. Following incubation, 500 µl of stop solution, 500 µl of color stabilizer 2 and 500 µl of color stabilizer 3 were added to each sample. Absorbance was measured at 550 nm using a plate reader (ELx808 Absorbance Microplate Reader, BioTek, Winooski, VT). LPS concentrations in Sigma-OVA and Hyglos-OVA were determined using standard curves obtained from different concentrations of LPS (0.1 EU/ml, 0.04 EU/ml, 0.02 EU/ml, 0.01 EU/ml, 0.005 EU/ml). No LPS was detected in the negative control.

### Animal preparation

Male BALB/c mice were obtained from Charles River Laboratories (Saint-Constant, Quebec, Canada) and Charles River Laboratories (Margate, UK) and were studied between the ages of 6–8 weeks. DO.11.10 mice, ovalbumin specific TCR transgenic mice with BALB/c background, and *TLR4* deficient (BALB/c background) mice were bred in the animal care facility of McGill University Health Centre. All animals were housed in conventional animal facilities at McGill University and University College Cork and were cared for in compliance with the Canadian Council of Animal Care’s guide; protocols and procedures were approved animal ethics committees of McGill University, Montreal, Canada and University College Cork, Ireland.

### Allergic sensitization and challenge protocol

The sensitization and challenge protocol used in this study is shown in supplementary figure 1. Briefly, all groups were sensitized with an i.p. injection of a phosphate-buffered saline (PBS) (Invitrogen, Paisley, Scotland or USA) solution containing 10 µg OVA (Grade V, Sigma) with 1 mg of adjuvant aluminum hydroxide, Al(OH)_3_ (Fisher Scientific, Ottawa, ONT, Canada). Sensitization was performed on two separate days; day 0 and day 7. One week following the second sensitization, mice were challenged once or on three consecutive days via an intra-nasal instillation of 10 µg of LPS-rich OVA or LPS-free OVA in 50 µl of saline under light isoflurane anaesthesia. One day after the single challenge and two days following the three challenges, experiments were performed. Control mice were challenged with PBS.

**Figure 1:**
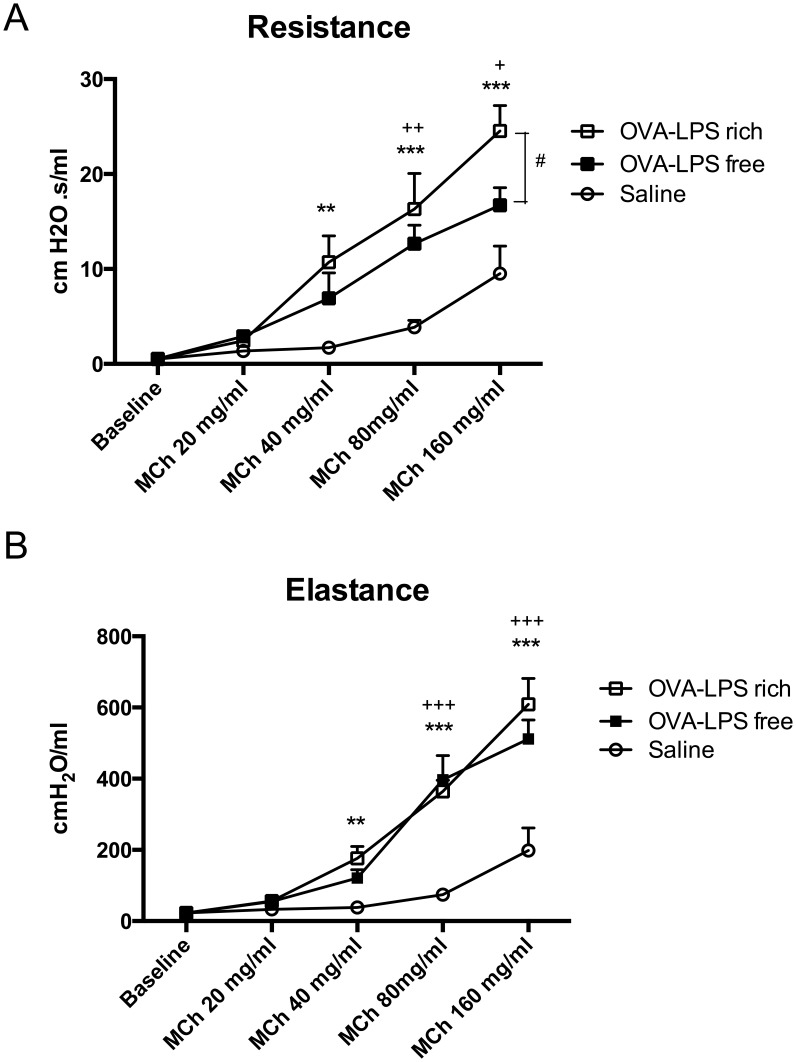
Contaminating LPS increases airway resistance to OVA following metacholine challenge. (a) Lung resistance in LPS-rich and LPS-free OVA challenged mice 48 hours following three OVA challenges, LPS rich OVA increased Lung resistance. (b) Lung elastance compared to saline control in both LPS-rich and LPS-free OVA challenged mice as per (a). Mice were sensitized with OVA on day 0 and 7 and challenged intra-nasally with either LPS-free or LPS-rich OVA on day 14, 15, 16 and assessed for lung mechanics on day 18. * OVA-LPS rich vs. PBS, + OVA-LPS free vs. PBS, # OVA-LPS free vs. OVA-LPS rich. Number of symbols denotes signficance, e.g, *** = P<0.001, Values are shown as Mean ± SEM, N = 8. Statistical significance was determined using ANOVA.

### Measurement of in vivo respiratory mechanics

Measurements of mechanics were made after three challenges only, as airway hyperresponsiveness (AHR) is inconsistently induced by a single challenge only. For this purpose, mice were injected i.p. with xylazine (10 mg/kg) and pentobarbital sodium (30 mg/kg). Mice were tracheostomized and an 18-gauge tracheal metal cannula was inserted and firmly tied. Subsequently, the animal was connected to a computer controlled small animal ventilator (Flexivent, Scireq Inc. Montreal, Canada) and normal tidal breathing was initiated. Mice were mechanically ventilated with a tidal volume of 10 ml/kg at a PEEP of 3 cm H_2_O and a frequency of 150 breaths/minute. Animals were then paralyzed with pancuronium bromide (1.2 mg/kg), administered i.p., prior to the measurement of respiratory mechanics.

Respiratory mechanics were measured using the *flexiVent* (SCIREQ Inc., Montreal, Canada) small animal mechanical ventilator. Briefly, total respiratory system resistance (Rrs) and elastance (Ers) were calculated by fitting the equation of motion of the linear single compartment model of lung mechanics to the data recorded from a 1.2 second, 2.5 Hz single-frequency forced oscillation perturbation, using multiple linear regression. Measurements were obtained at baseline as well as after the delivery of aerosols of increasing concentrations (20, 40, 80, 160 mg/ml in PBS) of methacholine (MCh; Sigma). Aerosols were delivered to the airways via an ultrasonic nebulizer for a period of 5 seconds (Aeroneb Lab, Aerogen Ltd, Ireland). Perturbations were executed every 8 seconds until the peak Rrs and Ers was observed for a given dose of MCh, and the process was repeated for each subsequent dose. The average of these values (peak Rrs and Ers) for each experimental group was then plotted.

### Bronchoalveolar lavage

Bronchoalveolar lavage (BAL) was performed immediately following single OVA challenge or, in the case of the three OVA challenges, following mechanics measurements. BAL was carried out using two aliquots of cold saline (1 ml) via the tracheal cannula. Supernatant from the first BAL sample for each animal was stored at −80°C for subsequent cytokine/chemokine analysis. Cell pellets were pooled and reconstituted in PBS for the determination of total viable cell counts and for cytospin preparations (Shandon, Cheshire, UK). Cytospins were stained with Diff-Quick (Fisher Scientific). Differential cell counts were then determined from a count of three hundred cells.

### Flow cytometric analysis of lung T helper and regulatory cells

We assessed the CD4+ T cell numbers and CD4+CD25+FoxP3+ cells in the lungs of animals undergoing three OVA challenges. Following the assessment of methacholine responsiveness and BALF collection, the right lung of each animal was excised and placed in cold RPMI-1640 medium, containing 8% heat-inactivated FBS, 2 mM L-glutamine, 50 µg/ml gentamycin and 10 mM HEPES (Invitrogen, USA.). The lung was removed from the medium and transferred to a dish containing 4 ml of sterile DPBS (Invitrogen) supplemented with collagenase (0.2 Wunsch units/ml, *Clostridium histolyticum*, Type XI-S, Sigma), DNAse I (1000 DNase units/ml, Type II-S, Sigma) and 0.5 mM calcium. The lung was also inflated with 1 ml of the same solution, minced with forceps and a scalpel blade and incubated on an orbital shaker at 37°C for 1 hour. The reaction was stopped with 5 ml cold RPMI-1640 medium (as above) with 2 mM EDTA (Invitrogen,) and 50 µM β-mercaptoethanol (Sigma). The lung tissues were mechanically disrupted, passed through 70 µm and 40 µm BD Falcon cell strainers and finally centrifuged at 1500 rpm for 5 min. Red blood cells were lysed with ammonium chloride and leukocytes were counted using a Beckman Coulter A^c^.T Counter. Cells were incubated with mouse BD Fc Block (BD Biosciences, Canada), then stained with FITC-conjugated rat anti-mouse CD4 mAb (clone H129.19), followed by PE CD25 (clone PC61), or the appropriate isotype control Ab (BD). Cells were then fixed with BD Cytofix/Cytoperm solution, incubated with 1% BSA in BD Perm/Wash solution and finally stained with APC Foxp3 mAb (clone FJK-16s) or isotype control Ab (eBioscience, San Diego, CA, USA). 50000 events were acquired for each condition using the BD FacsCalibur (Becton Dickinson). Cells were gated first based on CD4-positivity (vs side scatter) and then based on forward scatter (vs side scatter) to exclude debris or small dead cells.

### Splenocyte culture and OVA stimulation

Spleens from 8–10 week DO.11.10 mice were harvested and a single cell suspension was prepared as described for lung tissue above. Splenocytes were cultured in 24-well plates, 5×10^6^ cell/ml, for 3 days in RPMI 1640 supplemented with 10% FBS and penicillin, 100 I.U/ml, streptomycin, 100 ug/ml, L-glutamine, 2 mM, (all reagents were purchased from Invitrogen), with either OVA–LPS free or OVA-LPS rich and with or without TLR-4 inhibitor (TAK-242, InvivoGen, Cedarlane labs, Ontario, Canada). Cells were analyzed by flow cytometry as described above.

### Isolation and stimulation of human peripheral blood mononuclear cells

Human peripheral blood mononuclear cells (PBMC) were isolated from 6 healthy human male volunteers with the approval of the Ethics Committee of University College Cork. Nine mls of venous blood was isolated in sterile EDTA vacutainers (BD). The blood was mixed with an equal volume of PBS (Invitrogen), layered over 20 mls of Histopaque (Sigma) and centrifuged for 400 *g* for 30 minutes. The buffy coat was removed from the interface and washed twice by centrifugation (10 min at 300 *g*) using DMEM (Invitrogen), 10% fetal calf serum (Sigma) and 1% penicillin-streptomycin (Invitrogen). PBMC viability and cell number was evaluated using the Countess automated cell counter (Invitrogen) and were re-suspended in complete media at 1×10^6^ cells/ml. PBMCs were stimulated for 20 hrs with ultrapure LPS (100 ng/ml) (E coli 0111:B4, InvivoGen), LPS-rich OVA (100 ng/ml) and LPS-free OVA (100 ng/ml). Supernatants were collect for cytokine analysis.

### Bronchoalveolar Lavage Fluid (BALF) and PBMC supernatant cytokine assay

IFN-γ, IL-1β, IL-2, IL-4, IL-5, KC (CXCL1), IL-10, IL-12p40 and TNF-α levels were quantified using an electro-chemiluminescence multiplex system Sector 2400 imager from Meso Scale Discovery (MSD) (Gaithersburg, MD, USA) where antibodies labelled with a Sulfo-tag emitted light upon electrochemical stimulation. MSD ultra-senstitive kits for murine (BALF) or human PBMC samples were used respectively.

### Statistical analyses

The experiments performed on wild type BALB/c mice characterizing inflammatory responses to OVA sensitization and challenge were reproduced in both the laboratories of the investigators in Ireland and Canada. The data presented in the manuscript are a representative dataset. The experiments characterizing respiratory mechanics and those on the TLR4 deficient and the DO.11.10 mice were performed in Canada only. Experiments were usually performed over several days with all experimental groups represented on any given day.

Data are expressed throughout as the mean +/− standard error of the mean. Comparison among means was performed using ANOVA with Tukey’s post hoc test for multiple comparisons. For lung mechanics ANOVA with Bonferroni’s multiple comparison test was used. When data were not normally distributed the analyses were performed on log transformed data.

## Results

### Commercial OVA contains high level of endotoxin

LPS content of both OVA samples was determined using the Limulus Amebocyte Lysate (LAL) agglutination single test vial to qualitatively test for the presence of LPS in the two commercial sources of OVA that we explored. Using this test we demonstrated the presence of LPS in the Sigma-OVA (LPS-rich OVA) whereas there was no LPS detected in the Hyglos-OVA (LPS-free). To obtain a more quantitative assessment of LPS levels we used the LAL Chromogenic Assay and we found that LPS-rich OVA contained on average 400 ng endotoxin/10 **µ**g OVA, while LPS-free OVA had undetectable levels (Figure S1). Four different batches of Sigma-OVA were tested for endotoxin and the results demonstrated a range of concentrations from 75****ng to 1415 ng/10** µ**g OVA.

### AHR after OVA challenge is minimally further increased by LPS-rich OVA

The effects of LPS-rich OVA and LPS-free OVA on the induction of AHR to MCh were evaluated in animals that were challenged with OVA on three consecutive days and had MCh challenge two days later (Figure S2). Respiratory resistance (R_RS)_ was significantly greater in LPS-rich OVA challenged mice following administrations of MCh in concentrations of 40, 80 and 160 mg/ml and also in LPS-free OVA challenged mice at 80 and 160 mg/ml concentrations of MCh, in comparison with PBS challenged controls (Fig. 1*A*). Animals challenged with LPS-free OVA only differed in their responsiveness (R_RS_) at the highest MCh concentration (160 mg/ml) compared to LPS-rich OVA challenged mice (16.71±1.86 vs. 24.54±2.67 cm H_2_O.s/ml respectively).

Total respiratory elastance (E_RS_) was also significantly greater in LPS-rich OVA challenged mice at MCh 40 mg/ml, 80 and 160 mg/ml and LPS-free OVA challenged mice (at 80, and 60 mg/ml) compared to PBS controls (Fig. 1*B*). However, in contrast to the R_RS_, E_RS_ responses of LPS-rich OVA and LPS-free OVA challenged groups were not different in response to increasing concentrations of MCh.

### Contaminating LPS induces neutrophilia and increases BALF cellularity in wild type but not TLR4 deficient mice

**Figure 2:**
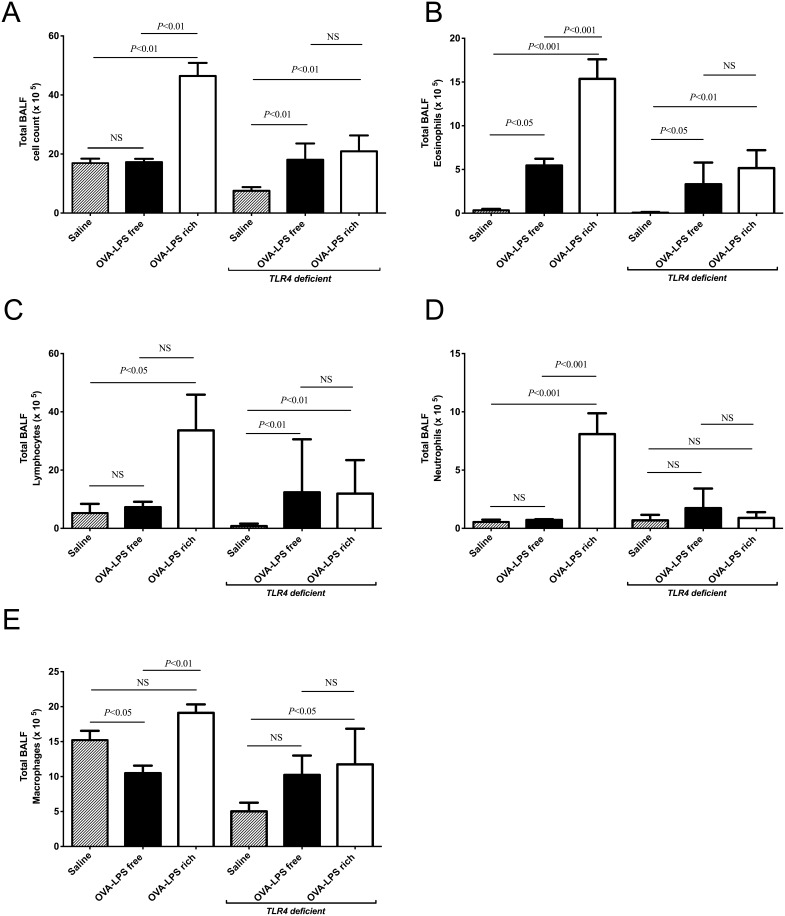
LPS contaminated OVA induces neutrophilia in bronchoalveolar lavage fluid (BALF) in wild type mice but not in *TLR4* deficient mice. Mice were treated as describe in Fig. 1, with three OVA challenges, and leukocytes were identified by morphological criteria and staining. (a) Total BALF cells in sensitized mice, following challenge with saline, LPS-rich OVA and LPS-free OVA. (b) Total eosinophils in BALF. (c) Total lymphocytes in BALF. (d) Total neutrophils in BALF. (e) Total macrophages in BALF. Values are shown as Mean ± SEM. (n = 8). Statistical significance was determined using ANOVA.

We assessed the differences in inflammatory response induced by OVA challenge at 24 hrs after a single OVA exposure and 48 hrs following three OVA challenges in both wild type and *TLR4* deficient mice. There was a significant increase in total cell counts in the BALF of LPS-rich OVA challenged animals compared to both the saline control and the LPS-free OVA in wild type animals (Fig. 2*A*). LPS-free OVA challenge did not significantly alter the total cell number. Challenge of *TLR4* deficient mice with LPS-free and LPS-rich OVA increased total cell counts (Fig. 2*A*). There was an increase in eosinophil numbers following challenge with both preparations of OVA but the LPS-rich OVA caused a relatively greater eosinophilia in wild type mice, (Fig. 2*B*). *TLR4* deficient mice also had increased eosinophil numbers from both OVA preparations, (Fig. 2*B*). In wild type mice LPS-rich OVA also evoked increases in neutrophil and lymphocyte numbers that were not seen with LPS-free OVA (Fig. 2*C, D*). Lymphocyte numbers were also increased in the *TLR4* deficient mice, but by both OVA types, however no change in neutrophils numbers was observed, (Fig. 2*C, D*). Macrophage numbers did not change between the LPS-rich OVA and saline challenged groups, however the macrophages were significantly fewer in the LPS-free OVA challenged group compared to saline and LPS-rich OVA challenge (Fig. 2*E*). LPS-rich OVA also induced an increase in macrophage numbers in *TLR4* deficient mice, (Fig. 2*E*), however LPS-free OVA did not.

### LPS-rich OVA induces a mixed Th2 and inflammatory response in the bronchoalveolar lavage

**Figure 3:**
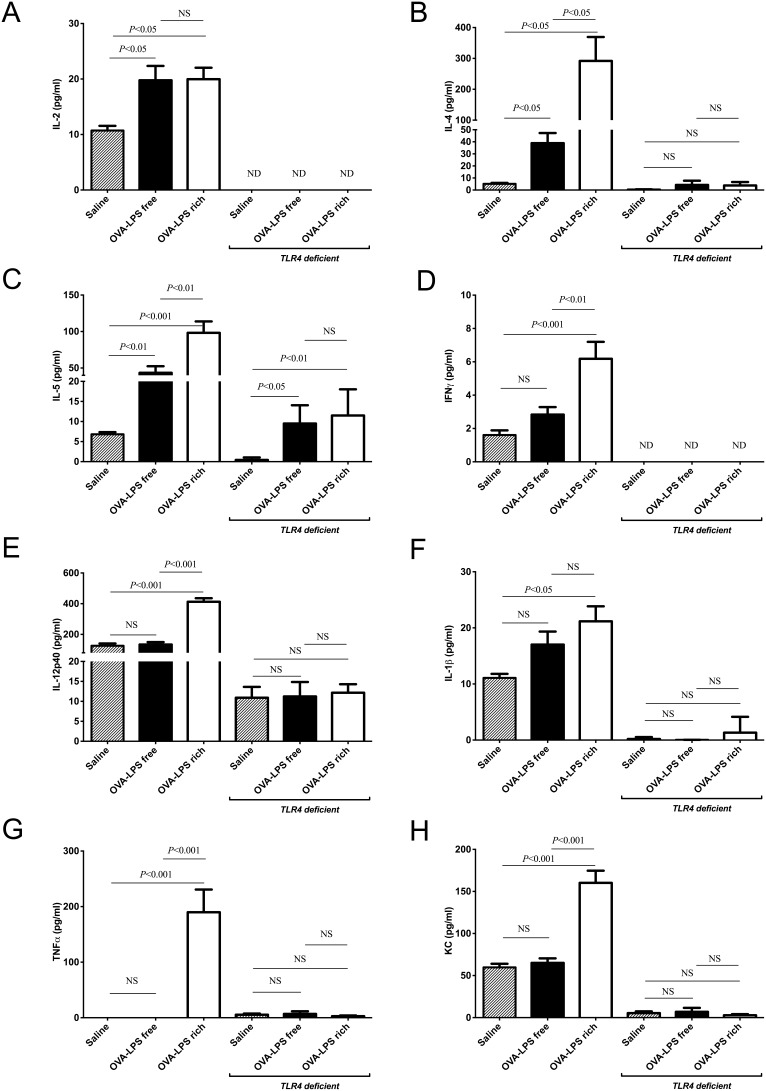
LPS contaminated OVA increase Th2 and Th1 cytokine levels in the BALF following nasal challenge in wild type mice but not in *TLR4* deficient mice. BALF cytokines were analysed using MSD electrochemical ELISA as described in the methods. Th2 cytokine levels secreted into the BALF of mice following three challenges with LPS-rich or LPS-free OVA, were assayed IL-2 (pg/ml) (a), IL-4 (b), IL-5 (c) and IFN-γ (d). Th1 cytokine levels secreted into the BALF of mice following three challenges with LPS-rich or LPS-free OVA, IL-12p40 (pg/ml) (e), IL-1β (f), TNFα (g) and KC (h). Not detectable (ND). Values are shown as Mean ± SEM. (n = 8). Statistical significance was determined using ANOVA.

The Th-2 cytokines, IL-2, IL-4 and IL-5, were increased in the wild type BAL fluid harvested at 24 hr after a single challenge of both groups with OVA (Fig. 3*A*–*C*). However the changes in IL-4 and IL-5 were greater in the group challenged with LPS-rich OVA compared to the LPS-free OVA challenged group. LPS-rich OVA also induced IFN-γ while LPS-free did not (Fig. 3*D*). LPS-rich OVA induced, in addition to the Th2 cytokines, increases in the inflammatory cytokines/chemokines; IL-12p40, IL-1β, TNF-α, and KC, the murine ortholog of IL-8, (Fig. 3*E*–*H*). No significant change in these mediators was observed in the LPS-free OVA challenged group. In the *TLR4*-deficient mice, no increase in IL-4 (Fig. 3B), was observed however IL-5 was induced to the same degree by the two OVA preparations (Fig. 3C). IL-2 and IFNγ were undetectable in the challenged *TLR4*-deficient mice BAL fluid. No increases in the inflammatory cytokines, IL-12p40, IL-1β, TNF-α, and KC were observed (Fig. 3*E*–*F*).

### OVA challenge increase helper T cells but only LPS-rich OVA increases Treg recruitment to the lung

**Figure 4:**
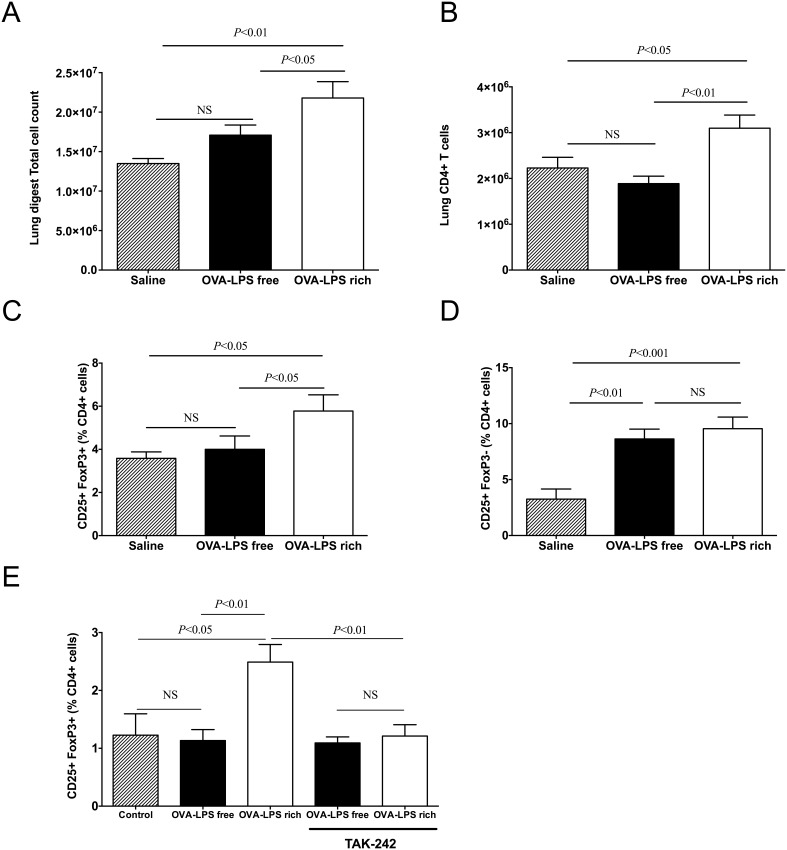
OVA challenge increases lung CD25+Foxp3− T (helper) cells while LPS-rich OVA also induces CD25+Foxp3+ T (reg) recruitment. Mice were treated as described in Fig. 1. Lung digests were performed, as described in the methods, cell populations were analysed by flow cytometry. (a) Total cell counts, (b) Total CD4+ T cell, (c) CD4+ CD25+Foxp3+ (Treg) cells and (d) CD4+CD25+ Foxp3− (activated T cells) in lung digests of sensitized mice following three challenges with saline, LPS-rich OVA and LPS-free OVA. (e) *in vitro* splenocyte Treg induction in the presence of the TAK-242 inhibitor with or without LPS-rich OVA and LPS-free OVA. Values are shown as Mean ± SEM. (n = 7). Statistical significance was determined using ANOVA.

Challenge of wild type mice with LPS-rich OVA, following three challenges, resulted in a significantly higher total lung cell count compared to both saline and LPS-free OVA (Fig. 4*A*). The total CD4+ cell numbers present in the lung were also elevated following LPS–rich challenge (Fig. 4*B*). The percentage of CD4+ cells that were CD25+ Foxp3+ in the LPS-rich challenged group was elevated and there was also an increase in the number of activated CD4+ T cells (CD25+ Foxp3−) (Fig. 4*C*). LPS-free OVA challenged mice did not have increased Tregs but did have increased activated helper T cells (CD4+CD25+) compared to saline (Fig. 4*D*). LPS-rich OVA also induced Treg differentiation *in* *vitro,* in splenocyte cultures from DO.11.10 mice, while LPS-free OVA did not; this effect was inhibited by adding the TLR4 inhibitor, TAK-242 (Fig. 4*E*).

### LPS-rich OVA induces an inflammatory cytokine response in Human PBMCs while LPS-free OVA does not

**Figure 5:**
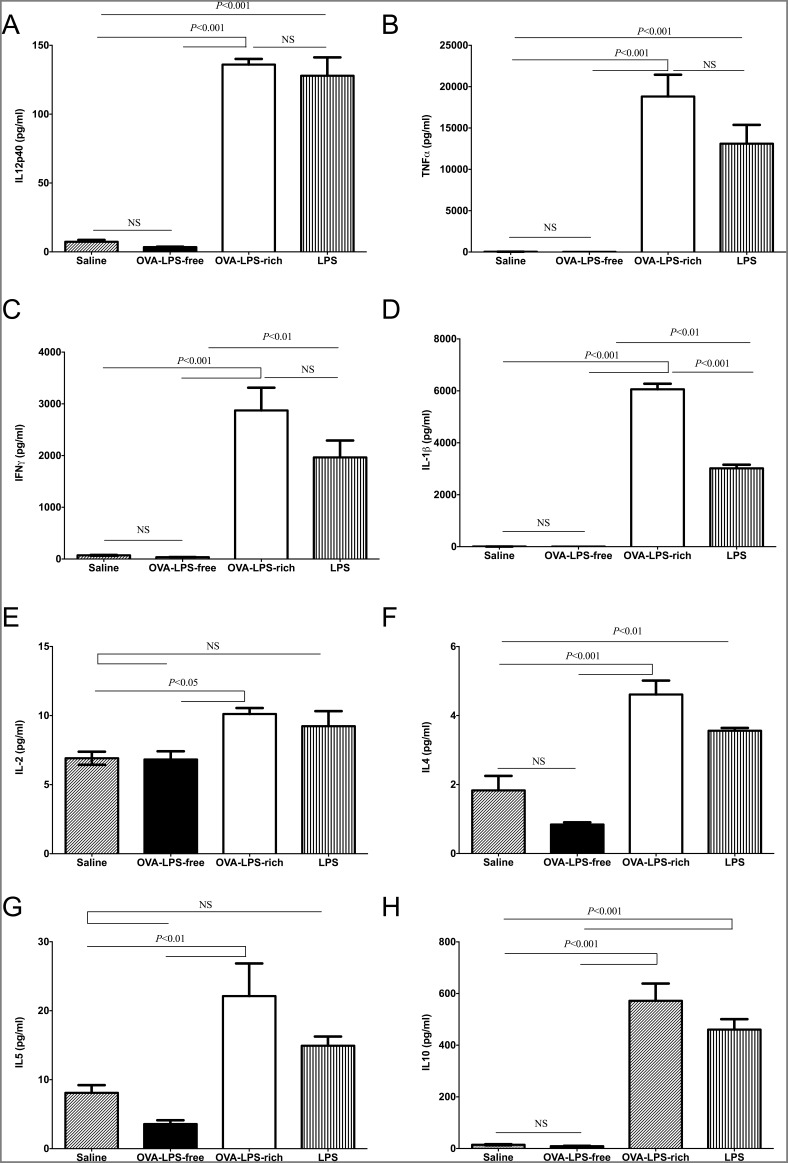
LPS-rich OVA induces a cytokine response in human PBMCs similar to LPS. PBMCs were stimulated for 20-free OVA, LPS-rich OVA and LPS, the supernatants were the analysed by MSD electrochemical ELISA for the levels of secreted cytokines, (a) IL-12p40, (b) TNFα (c) IFNγ, (d) IL-1β, (e) IL-2, (f) IL4, (g) IL-5 and (h) IL-10. Values are shown as Mean ± SEM. (n = 6). Statistical significance was determined using ANOVA.

It is possible that TLR4 on structural or immune effector cells might be responsible for conditioning T cell responses to LPS-rich OVA. Therefore we wished to determine whether the LPS-rich OVA evoked different responses in human PBMCs. Following incubation of PBMC for 20 hrs with either LPS-free OVA, LPS-rich OVA or control LPS (*E. coli* LPS, 100 ng/ml) the pro-inflammatory cytokines, IL-12p40, TNF-α, IFN-γ and IL1-β, were measured. LPS-rich OVA significantly induced all four Th1 cytokines with levels above that or comparable to the positive control LPS (Fig. *5A–D*). LPS-free OVA did not induce any cytokine response from the PBMCs. IL-2, IL-4, IL-5 and IL-10 levels were also increased by LPS-rich OVA but not LPS-free OVA (Fig. 5*E*–*H*).

## Discussion

There is abundant evidence that activation of the TLR pathway conditions subsequent responses to sensitization with soluble antigens. We evaluated the importance of LPS contamination in commercial OVA on the secondary inflammatory response evoked in a murine model of allergic asthma. There were substantial differences in the cellular and mediator responses to airway exposure to OVA in the presence and absence of LPS. In the context of sensitization using alum adjuvant, LPS modulates the inflammatory response, but is not necessary for “disease” development, as defined by the induction of airway hyperresponsiveness. Differences in inflammation induced by inhalational challenge with LPS-rich OVA and LPS-free OVA appear to be accounted for by signaling through the TLR4.

The endotoxin/LPS detected in the two commercial OVA preparations was evaluated using two assays, clot based and colorimetric. Both assays, although differing in sensitivity demonstrated the presence of endotoxin in the OVA that was not purified and its absence in the commercial LPS-free preparation. The LPS-rich OVA contained approximately 4 µg endotoxin/mg OVA. There was substantial batch-to-batch variation in the degree of endotoxin contamination. The levels of endotoxin detectable in relation to bacterial colony forming units (CFU) would suggest at least 2×10^6^ CFU of bacteria equivalents. [26] In a previous study Watanabe *et al.* [19] estimated the levels of endotoxin present in the same commercial source of OVA at 10 µg/mg, while our estimates indicated a comparable, substantial LPS contamination.

Airway challenge with LPS-rich OVA resulted in a substantially enhanced inflammation compared to challenge with LPS-free OVA. Both inflammatory and Th2-associated cytokines were augmented above the effects of OVA alone by the concomitant LPS challenge associated with commercial LPS-rich OVA. An increase in Tregs in lung digests was also observed following OVA challenge but only when associated with LPS. Despite the differences in inflammatory cells, cytokines and Tregs in the pulmonary response of LPS-rich OVA challenged animals, there was only a minor difference in the airway responses to inhaled aerosols of methacholine, and the difference was attributable to a slightly reduced degree of responsiveness of the larger conducting airways as reflected in respiratory system resistance. The changes were not detectable in respiratory system elastance, which is more sensitive to peripheral airway responses. A previous study [19] showing that endotoxin contamination of OVA reduced the hyperresponsiveness to methacholine induced by OVA in contrast to our findings but used the Penh technique, which is not a measure of airway resistance but rather reflects changes in breathing pattern that may or may not follow changes in respiratory mechanics. [27] Likewise, mice lacking lipopolysaccharide binding protein, an accessory molecule that is important for the affinity of LPS binding to TLR-4 [28], have reduced OVA induced airway responsiveness but again measured by the Penh technique [29]. A recent study on the BN rat undergoing repeated challenges with LPS-rich and LPS-free OVA demonstrated comparable airway smooth muscle remodeling in the large airways. [30].

Both OVA challenged groups showed substantial sensitization to OVA but there was, in general, an augmented inflammatory response to LPS-rich OVA challenge. An eosinophil-rich inflammation followed challenge in both OVA challenged groups but it was more pronounced after LPS-rich OVA exposure. Low dose LPS has been shown previously to augment eosinophilic inflammation in mice sensitized intranasally with LPS-free OVA. [20] Low dose systemic LPS has similar effects on pulmonary eosinophilia. [30] However, mice lacking lipopolysaccharide binding protein, reportedly mount comparable degrees of eosinophilia in response to OVA challenge to wild type controls, although they do not develop allergen-induced airway hyperresponsiveness. [29] However, the administration of exogenous LPS to OVA challenged C57Bl/6 mice is inhibitory of eosinophilia [20] as is high dose LPS challenge of BALB/c mice [25], indicating that the timing, route of administration, origin and dose of LPS may have important influences on the outcome of the challenge. In the current study there was also a neutrophilic response, a marked lymphocytosis and an increase in macrophage numbers in the LPS-rich OVA challenged mice, not seen in LPS-free OVA challenged wild type animals. The BALF neutrophilia was also absent in *TLR4*-deficient mice. These data suggest substantial differences in the synthesis and secretion of chemoattractant molecules, chemokines and lipid mediators in the OVA challenged groups, due to the presence of contaminating LPS. Our observations with the *TLR4*-deficient mice confirm the observations of Strohmeier *et al*. [29], that LPS is not needed for OVA to induce eosinophilia and also confirms that contaminating LPS exacerbates inflammation in the wild type animals.

The BALF cytokine mediator levels, in the wild type mice, were different in the two OVA challenged groups, consistent with the differences in cellular influx that we observed. There appeared to be similar levels of T cell activation as reflected in the levels of IL-2. However, the Th2 cytokines IL-4 and IL-5 were more highly expressed in the LPS-rich OVA challenged animals. Somewhat paradoxically the inflammatory cytokine IFN-γ was also increased in expression in the LPS-rich OVA challenged animals. There was also an increase in IL-12, presumably reflecting dendritic cell priming by LPS. [25], [31] Other inflammatory cytokines, IL1-β and TNFα were elevated only in the LPS–rich challenged group. Consistent with the neutrophilia seen in the BALF there was an increase in the chemokine KC, an ortholog of IL-8 that is a potent neutrophil chemoattractant. [32] It is possible that activation of the airway epithelium by LPS may have contributed to the elevation in KC. Activation of the nuclear factor kappa-light-chain-enhancer of activated B cells (NFκB) pathway in the airway epithelium has been reported following LPS challenge and is important in mediating LPS-induced airway neutrophilia. [33] Similarly, NFκB is activated within thirty minutes of OVA challenge [34], potentially as a result of the LPS present in the OVA. Both types of OVA induced the same degree of IL-5 in *TLR4*-deficient mice, whereas the IL-4, IL-1β, TNF-α, KC and IL-12p40 responses were blunted in *TLR4*-deficient mice, indicating that, in particular, the pro-inflammatory cytokine responses to OVA challenge are promoted by contaminating LPS and TLR4. The route of sensitization may also affect the immune response following challenge. [23] Airway sensitization with OVA and LPS results in a Th17 response while peritoneal sensitization promotes a strong Th2 cytokine mediated response [23] and the AHR produced is the result of synergy between IL-17 and Th2 cytokines. Th2 inflammation appears to have been the dominant cause of AHR in our study and the additional neutrophilic inflammation and augmented cytokines had only a modest effect on the changes in resistance and no effect on elastance in response to methacholine.

We evaluated the overall lung tissue T cell response, in wild type mice, by lung digestion and flow cytometry. Similar to the finding of increased total cells in BALF there was also an increase in total cells recovered from the lung digests after LPS-rich OVA challenge. In addition CD4+ T cells, Tregs and activated CD4+ T cells were all increased in the LPS–rich OVA challenged animals. Although there were comparable levels of IL-2 in BALF of both OVA challenged groups there was a greater absolute number of CD4+CD25+FoxP3− T cells present in the LPS-rich OVA challenged group. The increase in CD4+ T cells in the LPS-rich OVA challenged animals is also consistent with the higher levels of Th1 and Th2 cytokines in the BALF. Surprisingly there was an increase in Tregs only in the LPS-rich OVA challenged group indicating that Treg expansion or recruitment is facilitated by LPS. This effect was also observable *in vitro* when murine splenocytes were cultured with the LPS-rich OVA and could be prevented with the TLR4 inhibitor TAK-242. Despite the recruitment of Tregs to the lungs in animals challenged with LPS-rich OVA, the inflammatory response was still greater than in the LPS-free OVA challenged group. Although Tregs have been implicated in determining the intensity and the resolution of inflammation following pulmonary challenge with antigen. [35]–[37] they appear to have little role to play in the acute response to allergen challenge of a sensitized animal but are rather important for the development of tolerance to chronic allergen challenge. [38] Interestingly, Whitehead *et al*. recently reported that while the addition of a low-moderate concentration of LPS during intranasal OVA sensitization (comparable to the LPS concentrations found in ovalbumin in our study) promoted more substantial acute airway inflammation than lower levels of LPS, this concentration of LPS also induced Tregs and, after multiple allergen challenges, shorter-lived asthma-like features than in mice sensitized with lower doses of LPS [22]. Whereas LPS levels were modified during OVA sensitization but were equivalent during challenge, in our study, we demonstrate in identically sensitized animals that the induction of Foxp3-expressing Tregs is apparently entirely dependent on the contaminating LPS and TLR4 signaling associated with the inhalational allergen challenge, rather than the adjuvant effect of alum or LPS during intraperitoneal sensitization. Taken together, although it is not clear whether the Tregs in both studies are functionally equivalent, their expansion in response to inhaled antigen appears to depend on concomitant airway exposure to sufficient levels of LPS irrespective of whether this exposure coincides with sensitization or challenge. It is not clear whether the lack of resolution of airway disease and thus apparent absence of an inhibitory effect of the LPS-induced Tregs in our model may be due to a requirement for prior airway exposure to LPS and OVA (such as during sensitization), due to a need for more allergen challenges (premature experimental endpoint), higher dose of LPS and threshold of Tregs, or perhaps due to the alum-mediated pro-inflammatory response overcoming the inhibitory capacity of the induced Tregs. Finally, whether the induction of Tregs depends on TLR4 receptors on dendritic cells or T cells requires further exploration.

We tested also the response of human peripheral blood mononuclear cells (PBMC) to LPS-free and LPS-rich OVA to determine if a pattern of response specific to LPS-rich OVA was present *in vitro*. The LPS-free OVA did not induce any cytokine response whereas the LPS-rich preparation induced pro-inflammatory cytokines comparable to the effects of 100 ng of *E coli* LPS. It is interesting to note that both IFN-γ and IL-1β are significantly increased by the LPS-rich OVA even compared to pure LPS. MyD88-dependent signalling by both IFN-γ and LPS has been shown to induce steroid resistant AHR via IL-27 producing pulmonary macrophages. [39] IL-1β is released from the inflammasome upon Nod-like receptor stimulation by microbial molecules [40], of which LPS is one of many. This increased IFN-γ and IL-1β level would suggest that the commercial LPS-rich OVA may also contain other microbial-derived molecules which may induce inflammation and confound the immune response to the OVA allergen.

In the absence of adjuvant, stimulation of TLR4 on airway structural cells has been shown to be necessary for priming of innate immune responses and for the development of airway disease in response to inhaled house dust mite allergen (HDM). [41] Indeed HDM and LPS airway sensitization and challenge reduced Th2 responses and enhanced Th1 inflammatory responses, increasing both TNFα and IL-17 secretion. [42] Recently, Tan *et al.,* have also reported that TLR4 expression on stromal cells promotes Th2-biased allergic sensitization to OVA via the airways and the development of subsequent airway disease. [43] The levels of contaminating LPS in our study are comparable to the low-moderate level referred to by Tan *et al.* Here, we show that systemic OVA sensitization in conjunction with an exogenous adjuvant (alum) leads to AHR that is largely independent of the contaminating endotoxin and TLR4 signaling. However, our data reveal that although i.p. OVA sensitization with alum can elicit subsequent allergic airway disease independently of TLR4, as has been previously reported, this does not mean that the contaminating LPS in OVA and associated TLR4 signaling are devoid of any influence upon the ensuing allergen challenge-associated inflammatory response in an alum-dependent OVA-asthma model. Indeed, observations regarding the phenomenon of LPS tolerance by both Peters *et al* [44] and more recently by Chapman *et al*, [45] elegantly demonstrate the significant effect of repeated exposure to LPS and the respective LPS levels have on the cytokine levels and allergic lung response. LPS tolerance also results in long lasting effects on murine macrophage gene expression and phenotypes. [46] Therefore, our study provides a degree of refinement in our understanding of the mechanisms underlying such experimental asthma models. Specifically, in agreement with Tan *et al*., despite the sensitization with alum adjuvant, we find that the associated Th2 inflammatory response remains partially dependent on the contaminating endotoxin present with OVA allergen challenge. Significantly, the inflammatory cytokine, T regulatory cell, and neutrophil responses associated with OVA challenge are completely dependent on the presence of contaminating endotoxin and signaling via TLR4, and independent of the alum sensitization. We confirmed that these findings could be recapitulated with peripheral blood mononuclear cells.

In conclusion, LPS-contamination of commercial OVA is a confounding variable in the interpretation of studies of experimental asthma. The results of the current study show that the pattern and degree of inflammation in an alum-dependent OVA-asthma model, particularly neutrophil, lymphocyte and Treg responses, is substantially influenced by the presence of LPS specifically during the pulmonary antigen challenge.

## Supporting Information

Figure S1 **Timeline of OVA challenge comparing LPS-rich and LPS-free OVA preparations.** Mice were acclimated for 1 week prior to i.p. sensitization with 10 µg of OVA (Grade V, Sigma) in aluminum hydroxide. Sensitization was performed on Day 0 and 7, on day 14 mice were intransally challenged once or on three consecutive days with 10 µg of LPS-rich OVA (Sigma) or LPS-free OVA (Hyglos Gmbh). One day after the single challenge and two days following three challenges, experiments were performed.
(TIF)

Figure S2 **LAL assay analysis of endotoxin content in LPS-rich and LPS-free OVA.** LPS-rich OVA (Sigma) or LPS-free OVA (Hyglos) samples were diluted to determine the range of endotoxin concentration and samples were assessed using a chromogenic LAL assay. LPS-rich, Sigma grade V, contained 0.004% endotoxin, (400 ng/10 mg OVA), while LPS-free OVA, Hyglos-Endograde, had undetectable levels. LPS-rich OVA (light bars), LPS-free OVA (dark bars). Values are shown as Mean ± SEM, (n = 5).
(TIF)

## References

[1] Malo JL, Ghezzo H, D’Aquino C, L’Archeveque J, Cartier A, et al (1992) Natural history of occupational asthma: relevance of type of agent and other factors in the rate of development of symptoms in affected subjects. The Journal of allergy and clinical immunology 90: 937–944. 1460199 10.1016/0091-6749(92)90466-f

[2] Mosmann TR, Cherwinski H, Bond MW, Giedlin MA, Coffman RL (1986) Two types of murine helper T cell clone. I. Definition according to profiles of lymphokine activities and secreted proteins. Journal of immunology 136: 2348–2357. 2419430

[3] Hamid Q, Azzawi M, Ying S, Moqbel R, Wardlaw AJ, et al (1991) Interleukin-5 mRNA in mucosal bronchial biopsies from asthmatic subjects. International archives of allergy and applied immunology 94: 169–170. 1937868 10.1159/000235353

[4] Olivenstein R, Taha R, Minshall EM, Hamid QA (1999) IL-4 and IL-5 mRNA expression in induced sputum of asthmatic subjects: comparison with bronchial wash. The Journal of allergy and clinical immunology 103: 238–245. 9949314 10.1016/s0091-6749(99)70497-5

[5] Strachan DP (1989) Hay fever, hygiene, and household size. Bmj 299: 1259–1260. 2513902 10.1136/bmj.299.6710.1259 PMC1838109

[6] von Mutius E (2007) Asthma and allergies in rural areas of Europe. Proceedings of the American Thoracic Society 4: 212–216. 17607001 10.1513/pats.200701-028AW

[7] Holt PG, Upham JW, Sly PD (2005) Contemporaneous maturation of immunologic and respiratory functions during early childhood: implications for development of asthma prevention strategies. The Journal of allergy and clinical immunology 116: 16–24; quiz 25. 10.1016/j.jaci.2005.04.017 15990766

[8] Wills-Karp M, Luyimbazi J, Xu X, Schofield B, Neben TY, et al (1998) Interleukin-13: central mediator of allergic asthma. Science 282: 2258–2261. 9856949 10.1126/science.282.5397.2258

[9] Kung TT, Stelts DM, Zurcher JA, Adams GK 3rd, Egan RW, et al (1995) Involvement of IL-5 in a murine model of allergic pulmonary inflammation: prophylactic and therapeutic effect of an anti-IL-5 antibody. American journal of respiratory cell and molecular biology 13: 360–365. 7654390 10.1165/ajrcmb.13.3.7654390

[10] Ewart SL, Kuperman D, Schadt E, Tankersley C, Grupe A, et al (2000) Quantitative trait loci controlling allergen-induced airway hyperresponsiveness in inbred mice. American journal of respiratory cell and molecular biology 23: 537–545. 11017920 10.1165/ajrcmb.23.4.4199

[11] Corry DB, Folkesson HG, Warnock ML, Erle DJ, Matthay MA, et al (1996) Interleukin 4, but not interleukin 5 or eosinophils, is required in a murine model of acute airway hyperreactivity. The Journal of experimental medicine 183: 109–117. 8551213 10.1084/jem.183.1.109 PMC2192426

[12] Lambrecht BN (2005) Dendritic cells and the regulation of the allergic immune response. Allergy 60: 271–282. 15679711 10.1111/j.1398-9995.2005.00708.x

[13] Shainheit MG, Smith PM, Bazzone LE, Wang AC, Rutitzky LI, et al (2008) Dendritic cell IL-23 and IL-1 production in response to schistosome eggs induces Th17 cells in a mouse strain prone to severe immunopathology. Journal of immunology 181: 8559–8567. 10.4049/jimmunol.181.12.8559 PMC2663362 19050275

[14] Machida I, Matsuse H, Kondo Y, Kawano T, Saeki S, et al (2004) Cysteinyl leukotrienes regulate dendritic cell functions in a murine model of asthma. Journal of immunology 172: 1833–1838. 10.4049/jimmunol.172.3.1833 14734767

[15] Sokol CL, Barton GM, Farr AG, Medzhitov R (2008) A mechanism for the initiation of allergen-induced T helper type 2 responses. Nature immunology 9: 310–318. 18300366 10.1038/ni1558 PMC3888112

[16] Okayama Y, Petit-Frere C, Kassel O, Semper A, Quint D, et al (1995) IgE-dependent expression of mRNA for IL-4 and IL-5 in human lung mast cells. Journal of immunology 155: 1796–1808. 7543533

[17] Mosmann TR, Coffman RL (1989) TH1 and TH2 cells: different patterns of lymphokine secretion lead to different functional properties. Annual review of immunology 7: 145–173. 10.1146/annurev.iy.07.040189.001045 2523712

[18] Eisenbarth SC, Piggott DA, Huleatt JW, Visintin I, Herrick CA, et al (2002) Lipopolysaccharide-enhanced, toll-like receptor 4-dependent T helper cell type 2 responses to inhaled antigen. The Journal of experimental medicine 196: 1645–1651. 12486107 10.1084/jem.20021340 PMC2196061

[19] Watanabe J, Miyazaki Y, Zimmerman GA, Albertine KH, McIntyre TM (2003) Endotoxin contamination of ovalbumin suppresses murine immunologic responses and development of airway hyper-reactivity. The Journal of biological chemistry 278: 42361–42368. 12909619 10.1074/jbc.M307752200

[20] Dong L, Li H, Wang S, Li Y (2009) Different doses of lipopolysaccharides regulate the lung inflammation of asthmatic mice via TLR4 pathway in alveolar macrophages. The Journal of asthma: official journal of the Association for the Care of Asthma 46: 229–233. 19373628 10.1080/02770900802610050

[21] Piggott DA, Eisenbarth SC, Xu L, Constant SL, Huleatt JW, et al (2005) MyD88-dependent induction of allergic Th2 responses to intranasal antigen. The Journal of clinical investigation 115: 459–467. 15650773 10.1172/JCI22462 PMC544038

[22] Whitehead GS, Thomas SY, Cook DN (2013) Modulation of Distinct Asthmatic Phenotypes in Mice by Dose-Dependent Inhalation of Microbial Products. Environmental health perspectives. 10.1289/ehp.1307280 PMC3888577 24168764

[23] Wilson RH, Whitehead GS, Nakano H, Free ME, Kolls JK, et al (2009) Allergic sensitization through the airway primes Th17-dependent neutrophilia and airway hyperresponsiveness. American journal of respiratory and critical care medicine 180: 720–730. 19661246 10.1164/rccm.200904-0573OC PMC2778149

[24] Bortolatto J, Borducchi E, Rodriguez D, Keller AC, Faquim-Mauro E, et al (2008) Toll-like receptor 4 agonists adsorbed to aluminium hydroxide adjuvant attenuate ovalbumin-specific allergic airway disease: role of MyD88 adaptor molecule and interleukin-12/interferon-gamma axis. Clinical and experimental allergy: journal of the British Society for Allergy and Clinical Immunology 38: 1668–1679. 18631348 10.1111/j.1365-2222.2008.03036.x

[25] Eisenbarth SC, Colegio OR, O’Connor W, Sutterwala FS, Flavell RA (2008) Crucial role for the Nalp3 inflammasome in the immunostimulatory properties of aluminium adjuvants. Nature 453: 1122–1126. 18496530 10.1038/nature06939 PMC4804622

[26] KL W (2007) Endotoxins: pyrogens, LAL testing and depyrogenation. New York; London: Informa Healthcare.

[27] Lundblad LK, Irvin CG, Hantos Z, Sly P, Mitzner W, et al (2007) Penh is not a measure of airway resistance! The European respiratory journal. 30: 805. 10.1183/09031936.00091307 17906089

[28] Thomas CJ, Kapoor M, Sharma S, Bausinger H, Zyilan U, et al (2002) Evidence of a trimolecular complex involving LPS, LPS binding protein and soluble CD14 as an effector of LPS response. FEBS letters 531: 184–188. 12417309 10.1016/s0014-5793(02)03499-3

[29] Strohmeier GR, Walsh JH, Klings ES, Farber HW, Cruikshank WW, et al (2001) Lipopolysaccharide binding protein potentiates airway reactivity in a murine model of allergic asthma. Journal of immunology 166: 2063–2070. 10.4049/jimmunol.166.3.2063 11160257

[30] Tsuchiya K, Siddiqui S, Risse PA, Hirota N, Martin JG (2012) The presence of LPS in OVA inhalations affects airway inflammation and AHR but not remodeling in a rodent model of asthma. American journal of physiology Lung cellular and molecular physiology 303: L54–63. 22523281 10.1152/ajplung.00208.2011

[31] Wills-Karp M (2001) IL-12/IL-13 axis in allergic asthma. The Journal of allergy and clinical immunology 107: 9–18. 11149983 10.1067/mai.2001.112265

[32] Bozic CR, Gerard NP, von Uexkull-Guldenband C, Kolakowski LF Jr, Conklyn MJ, et al (1994) The murine interleukin 8 type B receptor homologue and its ligands. Expression and biological characterization. The Journal of biological chemistry 269: 29355–29358. 7961909

[33] Poynter ME, Irvin CG, Janssen-Heininger YM (2003) A prominent role for airway epithelial NF-kappa B activation in lipopolysaccharide-induced airway inflammation. Journal of immunology 170: 6257–6265. 10.4049/jimmunol.170.12.6257 12794158

[34] Poynter ME, Cloots R, van Woerkom T, Butnor KJ, Vacek P, et al (2004) NF-kappa B activation in airways modulates allergic inflammation but not hyperresponsiveness. Journal of immunology 173: 7003–7009. 10.4049/jimmunol.173.11.7003 PMC2830271 15557197

[35] Strickland DH, Stumbles PA, Zosky GR, Subrata LS, Thomas JA, et al (2006) Reversal of airway hyperresponsiveness by induction of airway mucosal CD4+CD25+ regulatory T cells. The Journal of experimental medicine 203: 2649–2660. 17088431 10.1084/jem.20060155 PMC2118157

[36] Morgan RK, McAllister B, Cross L, Green DS, Kornfeld H, et al (2007) Histamine 4 receptor activation induces recruitment of FoxP3+ T cells and inhibits allergic asthma in a murine model. Journal of immunology 178: 8081–8089. 10.4049/jimmunol.178.12.8081 17548646

[37] Lewkowich IP, Herman NS, Schleifer KW, Dance MP, Chen BL, et al (2005) CD4+CD25+ T cells protect against experimentally induced asthma and alter pulmonary dendritic cell phenotype and function. The Journal of experimental medicine 202: 1549–1561. 16314437 10.1084/jem.20051506 PMC2213331

[38] Curotto de Lafaille MA, Kutchukhidze N, Shen S, Ding Y, Yee H, et al (2008) Adaptive Foxp3+ regulatory T cell-dependent and -independent control of allergic inflammation. Immunity 29: 114–126. 18617425 10.1016/j.immuni.2008.05.010

[39] Li JJ, Wang W, Baines KJ, Bowden NA, Hansbro PM, et al (2010) IL-27/IFN-gamma induce MyD88-dependent steroid-resistant airway hyperresponsiveness by inhibiting glucocorticoid signaling in macrophages. Journal of immunology 185: 4401–4409. 10.4049/jimmunol.1001039 20817868

[40] Franchi L, Warner N, Viani K, Nunez G (2009) Function of Nod-like receptors in microbial recognition and host defense. Immunological reviews 227: 106–128. 19120480 10.1111/j.1600-065X.2008.00734.x PMC2679989

[41] Hammad H, Chieppa M, Perros F, Willart MA, Germain RN, et al (2009) House dust mite allergen induces asthma via Toll-like receptor 4 triggering of airway structural cells. Nature medicine 15: 410–416. 10.1038/nm.1946 PMC2789255 19330007

[42] Daan de Boer J, Roelofs JJ, de Vos AF, de Beer R, Schouten M, et al (2013) Lipopolysaccharide inhibits Th2 lung inflammation induced by house dust mite allergens in mice. American journal of respiratory cell and molecular biology 48: 382–389. 23239494 10.1165/rcmb.2012-0331OC

[43] Tan AM, Chen HC, Pochard P, Eisenbarth SC, Herrick CA, et al (2010) TLR4 signaling in stromal cells is critical for the initiation of allergic Th2 responses to inhaled antigen. Journal of immunology 184: 3535–3544. 10.4049/jimmunol.0900340 20194715

[44] Peters M, Dudziak K, Stiehm M, Bufe A (2010) T-cell polarization depends on concentration of the danger signal used to activate dendritic cells. Immunology and cell biology 88: 537–544. 20125117 10.1038/icb.2010.3

[45] Chapman TJ, Emo JA, Knowlden SA, Rezaee F, Georas SN (2013) Pre-existing tolerance shapes the outcome of mucosal allergen sensitization in a murine model of asthma. Journal of immunology 191: 4423–4430. 10.4049/jimmunol.1300042 PMC3796001 24038084

[46] O’Carroll C, Fagan A, Shanahan F, Carmody RJ (2014) Identification of a unique hybrid macrophage-polarization state following recovery from lipopolysaccharide tolerance. Journal of immunology 192: 427–436. 10.4049/jimmunol.1301722 24337373

